# Graves Disease with Exophtalmia in a Two-Year Old Child

**DOI:** 10.4274/MIRT.020058

**Published:** 2011-12-01

**Authors:** Hülya Yalçın, Bülent Akçora, Ali Balcı

**Affiliations:** 1 Mustafa Kemal University School of Medicine, Department of Nuclear Medicine, Hatay, Turkey; 2 Mustafa Kemal University School of Medicine, Department of Pediatric Surgery, Hatay, Turkey; 3 Mustafa Kemal University School of Medicine, Department of Radiology, Hatay, Turkey

**Keywords:** Graves disease, exophthalmia, early childhood

## Abstract

Thyrotoxicosis is one of the rare disorders diagnosed in childhood and adolescence. The most frequent cause is Graves disease. One of the Graves' disease complications is thyroid-associated orbitopathy. A 2-year-old girl was referred to our hospital for decreased weight gain. Her physical examination was normal except for a palpable thyroid tissue and exophtalmia. After laboratory examination, she was referred to the departments of nuclear medicine and radiology for the diagnosis of hyperthyroidism and Graves ophthalmia. When evaluated with the physical examination, laboratory and imaging results the patient was diagnosed as Graves' disease with orbital involvement. So the patient was taken on methimazole treatment.

**Conflict of interest:**None declared.

## INTRODUCTION

Normal child development is influenced by thyroid hormones. Nervous system myelination, growth and puberty, dental and skeletal development, metabolism and organ function regulations are controlled by thyroid hormones (1). Thyrotoxicosis is one of the rare disorders diagnosed in childhood and adolescence. The incidence of thyrotoxicosis in young children is 0.1 in 100.000, and the most frequent cause is Graves' disease. 2 Thyromegaly, hyperthyroidism and infiltrative ophthalmopathy are the characteristic clinical signs of Graves' disease. 3 This process is an autoimmune inflammatory process linked to Graves hyperthyroidism. Graves' disease and infiltrative ophthalmopathy are described for the first time in 1835 and are mostly seen in adults. Up to now there is no publication in literature emphasizing the occurrence of Graves' opthalmopaty in children under 3 years old (2).

## CASE REPORT

**Figure 1:**
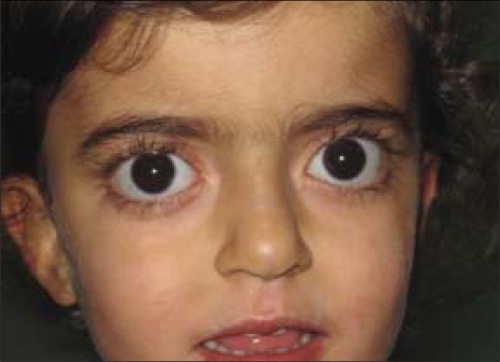
Photograph of the patient showing exophthalmia

A 2-year-old girl was referred to our hospital for decreased weight gain for one year. Her physical examination was normal except for a palpable thyroid tissue and exophthalmia (Figure 1). She was born at term and had no thyroid disorder at birth as well as no family history. Laboratory results showed that: free T3 20.35 pg/ml (normal limits: 2.5-3.9 pg/ml), free T4.

**Figure 2:**
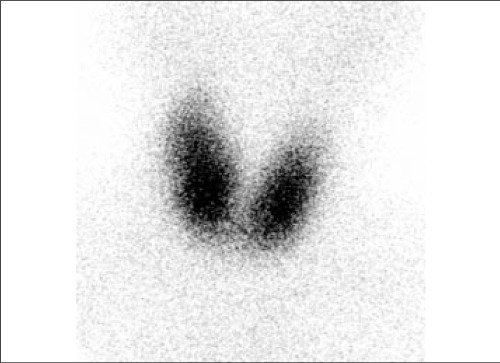
Tiroid scintigraphy shows bilateral minimal hyperplasia withincreased uptake

**Figure 3:**
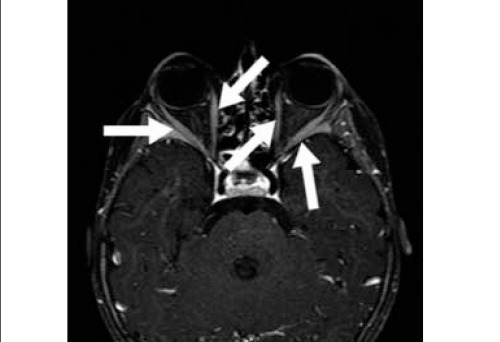
T1 weighted fat saturated contrast enhanced axial MR imageshows marked enhancement (arrows) of extraocular muscles of bilateral orbits

4.87 ng/dl (normal limits: 0.61-1.12 ng/dl), total T3 6.19 ng/dl (normal limits: 0.79-2.8 ng/dl), total T4 22.46 ug/dl (normal limits: 7-15.5 ug/dl), TSH 0.08 uIU/ml (normal limits: 0.5-6.5 uIU/ml),antimicrosomal antibody (AMA) 199.8 U/ml (normal limist: 0- 60 U/ml), antithyroglobulin antibody (ATA) 143.2. U/ml (normal limits: 0-120 U/ml). After physical and laboratory examination, she was referred to departments of nuclear medicine and radiology for thyroid scintigraphy and ultrasonographic evaluation. On ultrasonography, she had enlarged thyroid tissue with homogeneous parenchymal echogenicity. Thyroid scintigraphy was performed by 2 mCi of Tc-99m pertechnetate and imaging was performed 15 minutes later with Symbia-S dual head gamma camera (Siemens, Germany) equipped with a low energy-all purpose collimator. Increased uptake with minimally increased size in thyroid gland was seen in the scintigraphic images. (Figure 2). After the physical, laboratory and radiological examination, the patient is referred to magnetic resonance imaging for the confirmation of exophthalmia. Contrast-enhanced fat-saturated T1 weighted MR images showed bilateral orbital muscle involvement (Figure 3). When evaluated with the physical examination, blood tests and imaging results the patient was diagnosed as Graves' disease with orbital involvement. So the patient was taken on methimazole treatment.

## DISCUSSION

Graves' disease is a thyrotoxicosis associated with goiter, exophthalmia, tachycardia and hyperactivity. There are few cases reporting all of these signs in the literature. We observed Graves' ophthalmia in our case. Graves' ophthalmopathy in children and adolescents is thought to be a substantially benign process; its severity increases with age (4). It is also known that even though the hyperthyroidism is treated, significant problem is seen due to the ophthalmopathy because it leads to permanent cosmetic and functional sequelae, such as eyelid retraction, proptosis, keratopathy, compressive optic neuropathy, and strabismus. Many papers related to Graves' ophthalmopathy in adults are published in the literature, However, there are limited data about clinical features of Graves' ophthalmopathy in pediatric patients (5,6). There is not enough data that shows apparent infiltrative ophthalmopathy in children and adolescents. Milder ocular manifestations like retraction of the upper eyelid, upper lid lag and "staring eyes" are reported in ranging from 25 to 60% of all patients (7) The diagnosis is made with clinical examination and imaging tests, no laboratory test is available for the diagnosis of ophthalmopath (8). Diagnosis of hyperthyroidism in children is also very important due to brain development in this period. In the paper of Segni et al, they presented 3 cases with Graves' disease and one of them had a previous surgery for craniosynostosis before the diagnosis of Graves' disease. This patient was evaluated after her surgery and diagnosed as hyperthyroidism. After that period, she was not followed and one year later she applied to the hospital for psychomotor delay and poor nutrition. The other 2 cases were diagnosed as Graves' at 3 years of age and they were presented with the major signs of Graves' disease. All these patients were treated with antithyroid drugs (9). In our case, Graves' disease and ophthalmopathy are observed in a 2 year old girl. She had no brain sequelae, tachycardia, hyperactivity, delay of expressive language unlike the patients in literature. To our knowledge, there is no other case in the literature, who had scintigraphic evaluation and reported as Graves' ophtalmopathy under 3 years of age and diagnosed without prominent symptoms except the difficulty inweight gain. So the ophthalmic changes in the early childhood should be checked for hyperthyroidism.
